# *Listeria monocytogenes* Infection of Bat *Pipistrellus nathusii* Epithelial cells Depends on the Invasion Factors InlA and InlB

**DOI:** 10.3390/pathogens9110867

**Published:** 2020-10-22

**Authors:** Olga Povolyaeva, Yaroslava Chalenko, Egor Kalinin, Olga Kolbasova, Elena Pivova, Denis Kolbasov, Sergey Yurkov, Svetlana Ermolaeva

**Affiliations:** 1 Federal Research Center for Virology and Microbiology (FRCVM), 601125 Volginsky, Russia; 2741188@mail.ru (O.P.); olgakolbasova@gmail.com (O.K.); lenamail09@inbox.ru (E.P.); kolbasovdenis@gmail.com (D.K.); patronn13@rambler.ru (S.Y.); 2 Federal Research Center for Virology and Microbiology (FRCVM), Nizhny Novgorod Research Veterinary Institute Branch, Laboratory of Molecular Microbiology, 603022 Nizhny Novgorod, Russia; drermolaeva@mail.ru; 3 Gamaleya Research Center of Epidemiology and Microbiology, Laboratory of Ecology of Pathogenic Bacteria, 123098 Moscow, Russia; kalinin.egor@bk.ru

**Keywords:** *Listeria monocytogenes*, *Pipistrellus nathusii*, finite kidney cell lines of bat, invasion factors, InlA, InlB

## Abstract

*L. monocytogenes* is a widespread facultative intracellular pathogen. The range of natural hosts that supporting *L. monocytogenes* persistence in the environment has not been fully established yet. In this study, we were interested in the potential of *L. monocytogenes* to infect cells of bats, which are being increasingly recognized as a reservoir for microorganisms that are pathogenic to humans and domestic animals. A stable epithelial cell line was developed from the kidneys of *Pipistrellus nathusii*, a small bat widely distributed across Europe. The wild-type *L. monocytogenes* strain EGDe infected this cell line with an invasion efficiency of 0.0078 ± 0.0009%. Once it entered bat cells, *L. monocytogenes* doubled within about 70 min. When *L. monocytogenes* lacked either of the major invasion factors, InlA and InlB, invasion efficiency decreased by a factor of 10 and 25 respectively (*p* < 0.000001). The obtained results suggest that bat epithelial cells are susceptible to *L. monocytogenes* infection and that *L. monocytogenes* invasion of bat cells depends on the major invasion factors InlA and InlB. These results constitute the first report on in vitro studies of *L. monocytogenes* infection in bats.

## 1. Introduction

Bats are increasingly being recognized as reservoir hosts of microorganisms that can affect humans and domestic animals [1]. In the last several years, attention has primarily focused on highly pathogenic viruses, such as Marburg virus, Nipah virus, Hendra virus, Rabies virus, and coronaviruses hosted by bats [2,3,4,5,6,7]. Much less attention has been paid to pathogenic bacteria in bats. Nevertheless, available information supports the assumption that bats can host representatives of both novel pathogenic genera such as *Bartonella*, *Borrelia*, *Leptospira*, *Pseudomonas*, and *Acinetobacter* and well-known pathogens such as *Salmonella enterica, Escherichia coli, Yersinia pseudotuberculosis* and *Listeria monocytogenes* [8,9,10,11,12,13,14].

The majority of the listed bacteria were detected in the feces and gastrointestinal tracts of bats. The question of whether bats are just carriers of pathogenic bacteria or are themselves susceptible to infectious diseases needs to be elucidated for each causative agent [1,15]. There are only a few reports of serious diseases in bats caused by human pathogens. Lethal outbreaks caused by *Y. pseudotuberculosis* in flying foxes and *Egyptian rousette* bats have been reported [12,16,17]. Given the fact that bats are one of the most abundant animals on the planet, it can be supposed that their involvement as susceptible hosts in the epidemiology of human and animal pathogens circulating in natural ecosystems might substantially contribute to maintaining their pathogenic potential and transferring to human populations [18]. Features of bat ecology and physiology, such as the colonial character of life, movement of individuals on relatively long distances, roosting in proximity to urban and agricultural landscapes or even within human dwellings houses, demonstrate the potential for bats to provide circulation of highly humans virulent strains of bacterial pathogens between natural foci reservoirs of infection and human habitat [19,20,21,22,23].

The Gram-positive bacterium *L. monocytogenes* causes listeriosis, a severe disease in humans and a wide range of animals [24]. *L. monocytogenes* is widely distributed in nature and has been isolated from many wild animal species, including small rodents, deer, wild boar, wild birds, fish, and bats [14,25,26,27,28,29]. The spreading of *L. monocytogenes* clones, which are highly virulent for humans, in wildlife makes the natural reservoirs of listeriosis a serious concern [27,30]. In natural conditions, *Listeria* infection in animals and humans occurs mainly via contaminated food, but can occur through the nasal cavity, conjunctiva of the eye, respiratory organs, and damaged skin [31,32,33,34,35,36,37,38] [31,32,33,34,35,36,37,38]. The main sources of infection in listeriosis are sick animals that release the pathogen into the external environment with feces, urine, milk, and effluents from the nasal cavity and the genitals. Carriers of *L. monocytogenes* are widespread among mammals also support the spread of *L. monocytogenes* [39].

Reported isolation of *Listeria* spp. from bats confirms the ability of *L. monocytogenes* to be transmitted by bats [8,14]. The question of whether bats are susceptible hosts for *L. monocytogenes* is less studied. Isolation of *L. monocytogenes* from bat intestinal lymph nodes suggests a potential for the development of listeriosis in bats, because crossing the intestinal barrier and entering the intestinal lymph nodes are the first steps in the development of generalized infection caused by *L. monocytogenes* [14,24]. 

*L. monocytogenes* is a facultative intracellular pathogen, and the key point in the crossing of the intestinal barrier and the development of listeriosis is the active invasion of bacteria into host cells [24,40]. The virulence factors InlA and InlB are responsible for the active invasion of *L. monocytogenes* in mammalian non-phagocytic cells, i.e., epithelial, endothelial, and parenchymal cells [41,42]. The host receptor of InlA is the epithelial cell receptor E-cadherin [43]. InlB interacts with the tyrosine kinase c-Met and with the complement system receptor gC1q-R [44,45,46]. Interactions between InlA and E-cadherin are of key importance for the efficient crossing of the intestinal barrier [47]. InlB promotes *Listeria* invasion of M-cells located in Peyer’s patches and improves the crossing of the intestinal barrier [48,49,50].

Laboratory mice and guinea pigs are the most convenient laboratory models for studying listeriosis [51]. Although studying *Listeria* infection in the mouse model has led to the discovery of the majority of *L*. *monocytogenes* virulence factors [52], this model has some limitations, and murine listeriosis does not have all the characteristics of human listeriosis. In mice, oral administration of *L*. *monocytogenes* is not very efficient, and bacteria do not appear to have a selective tropism for the central nervous system and the fetoplacental unit [53]. The low ability of *L*. *monocytogenes* to cross the epithelial barriers of murine cells is in part due to the lack of interactions between the *Listeria* invasion protein InlA and mouse E-cadherin [53]. *Listeria* invasion protein InlB is inefficient in guinea pigs [54]. An alternative laboratory model for studying listeriosis is gerbils, which are small rodents that inhabit Asia, India, and Africa [55]. In gerbils, as in human listeriosis, both InlA and InlB are required for active *Listeria* invasion of a cell [53].

Given the wide range of hosts, as well as their different pathogenicity in previously studied model organisms, it becomes apparent that understanding the processes of *L. monocytogenes* evolution and selection of hypervirulence clones is impossible without knowing the mechanisms underlying the virulence of *L. monocytogenes* for different groups of mammals.

Discovering *L. monocytogenes* in the bat’s intestinal lymph nodes suggested that they are susceptible hosts of this pathogen [14]. However, the assessment of the intracellular potential of *L. monocytogenes* in bats has not been conducted to date. Moreover, there are no data on whether both major *L. monocytogenes* invasion factors—InlA and InlB—are necessary for *Listeria* invasion of bat cells. In this work, we address these questions and assess a new cell model for studying the mechanisms of pathogenicity of *L. monocytogenes.*

## 2. Results

### 2.1. Primary Cell Cultures and Their Characteristics

**Figure 1:**
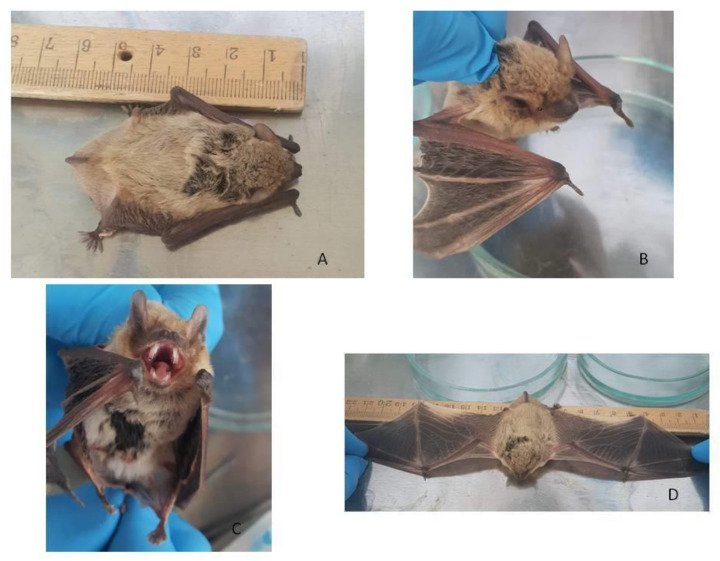
Morphological characteristics of *P. nathusii* caught in the Volgograd region. (**A**) Forest bat (lat*. Pipistrellus nathusii*), male. *P. nathusii* is a small bat from the family of *Vespertilionidae* bats, with adults reaching a length of 6 cm and body weight of 11 grams; (**B**) The photo shows a dense coat of medium length; the base of the hairs is dark, the color of the back is reddish-brown, and the abdomen is paler and grayish-yellow; the ear is relatively large, pointed, with tragus elongated with a rounded tip; the thumb is longer than the width of the wrist on the folded wing; (**C**) The photo shows that the outer upper incisor is slightly shorter than the inner one; (**D**) The wingspan of an adult is 25 cm. All of this corresponds to this type.

Nathusius’ pipistrelle (forest bat, *Pipistrellus nathusii*) is a small bat widely distributed across Europe [56]. In particular, it is a typical inhabitant of forests in the European regions of Russia. The length of this bat (head and body) reaches 60 mm with a wingspan of about 220–250 mm. Its weight is about 12–16 g [57]. Two typical male individuals captured in the Volgograd region, Russia, were used as donors of kidney epithelial cells (Figure 1).

**Figure 2:**
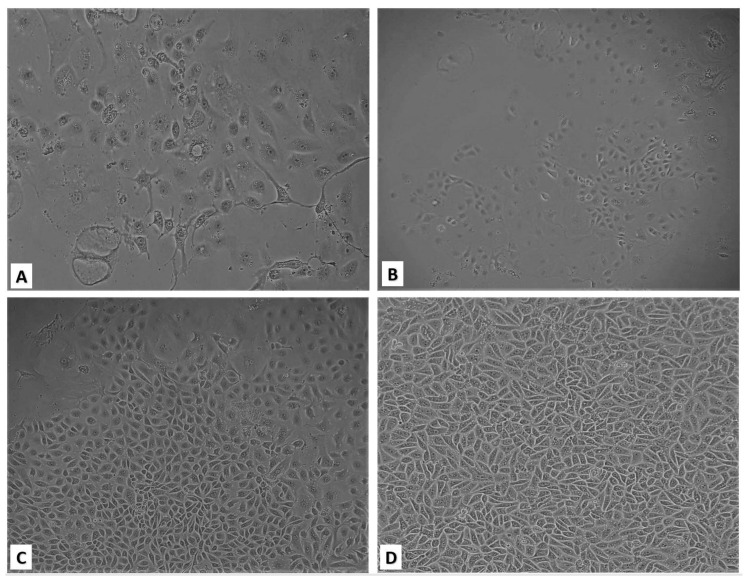
Bat kidney cell culture (magnification ×100). (**A**) Monolayer of primary culture presented morphologically heterogeneous cellular subpopulations with a predominance of epithelial-like cells; (**B**) Day 3 after seeding: A small number of cells formed clusters; (**C**) Day 4: Propagated cell clusters; (**D**) Day 5: Confluent proliferated monolayer.

Primary culture obtained from trypsinized kidney tissue was represented by morphologically heterogeneous cellular subpopulations with the predominance of epithelial-like cells (Figure 2A). After the first re-plating, cells showed islet growth at 72- and 96-hour post-plating. On the 5th day, islands merged to form a confluent monolayer (Figure 2B–D).

Upon the formation of a confluent monolayer of primary cells, subsequent re-plating steps were performed 2–3 times a week. At a seeding concentration of 1.5 × 10^5^ cells/mL, the formation of a confluent monolayer took 72 h, and the proliferation index was 3.3. The cell monolayer showed no signs of cell degeneration, and the cytopathic effect was preserved without changing the medium for 45 days (observation period).

Morphological research of subcultures showed that starting from the fourth passage, the monolayer consisted of epithelial-like cells with a polygonal shape. Their nuclei were oval or ellipsoid, less often spherical, with 1–3 (sometimes more) spherical nucleoli varying in size. The nuclear matrix was uniform. When passaging continued, the cell monolayer retained its characteristic morphology. The cell culture was designated as bat kidney epithelial cells (bKEC). The viability of bKEC cells before cryopreservation was 98%, and recovery after thawing ranged from 79% to 85%. bKEC cells were stable and able to survive in low-temperature storage for more than a year (data not shown). 

**Figure 3:**
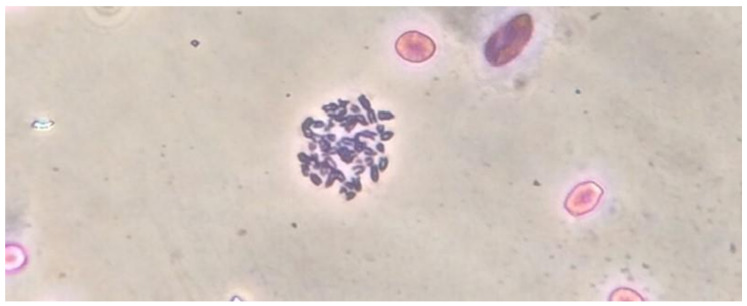
Cytogenetic analysis of *P. nathusii* cells. For the preparation of chromosome analysis, a daily culture of bat kidney epithelial cells (bKEC) cells was utilized. Cytogenetic analysis was performed using 50 metaphase plates of cell culture (magnification ×1000).

Cytogenetic analysis was performed to confirm the species identity of cells and to assess the stability of their chromosome set under long-term cultivation conditions. The cytogenetic analysis of 50 metaphase plates of bKEC subcultures at the second and third passages revealed a diploid modal number of 2n = 44 and a total number of chromosomal arms of FN = 50 before and after thawing (Figure 3). Cytogenetic analysis of the bKEC subculture at the 36th passage demonstrated the stability of the karyotype, which preserved both the diploid set of chromosomes and the absence of chromosomal rearrangements and the formation of marker chromosomes. Thus, our results confirm that the obtained culture of kidney cells of the bat *P. nathusii* retains the diploid set of chromosomes for at least 36 passages (the observation period).

### 2.2. L. monocytogenes Invades and Reproduces in Bat Kidney Cells

**Figure 4:**
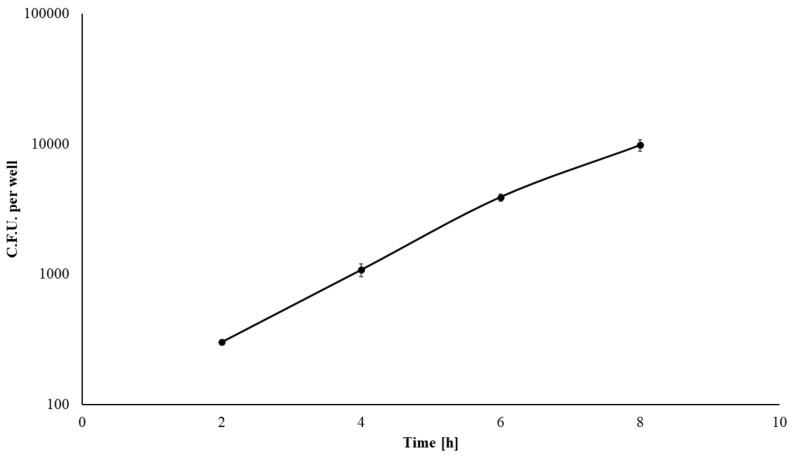
*L. monocytogenes* EGDe replication in bKEC cells. Approximately 3.8 × 10^4^ eukaryotic bat kidney epithelial cells were infected with a multiplicity of infection (MOI) of 100, and the numbers of intracellular bacterial cells were determined 2, 4, 6 and 8 hours post-infection. Mean ± SD from three experiments conducted in triplicate are shown.

The bKEC subculture taken at the 4th passage was infected with the wild-type *L. monocytogenes* strain EGDe. We used a standard gentamicin assay to infect 70% bKEC monolayer with *L. monocytogenes* at a multiplicity of infection (MOI) of 1:100 (cell/bacteria). Two hours post-infection, the average number of intracellular bacteria was 301 ± 11 CFU per well (Figure 4). The invasion efficiency measured as the percentage of intracellular bacteria relative to applied bacteria was 0.0078 ± 0.0009%. Despite the relatively low invasion efficiency, once inside bat cells, *L. monocytogenes* multiplied effectively, with a doubling time of 70 min. Thus, the number of intracellular bacteria increased more than 32-fold within 8 hours post-infection (Figure 4). The obtained results suggested that bat primary cells are susceptible to *L. monocytogenes* infection. 

### 2.3. L. monocytogenes Cell-to-Cell Spread

**Figure 5:**
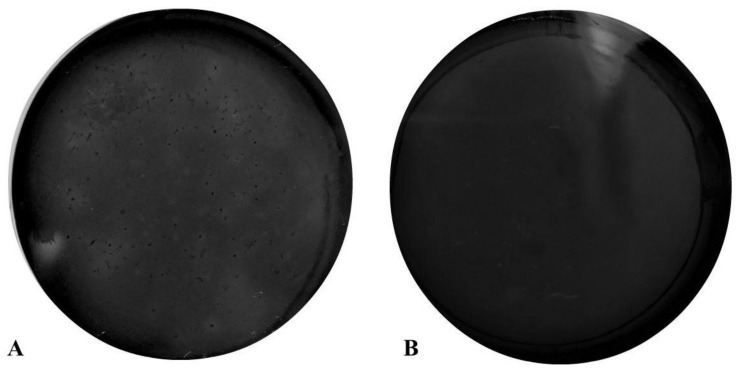
Plaques formed by *L. monocytogenes* in the monolayer of the bat kidney epithelial cells. (**A**) Images of *L. monocytogenes* EGDe plaques spreading through a monolayer of bKEC for 72 h in a 6-well plate; (**B**) A monolayer of uninfected cells as a control.

To further investigate the process of intracellular parasitism of *L. monocytogenes* we studied bacterial spreading. The effectiveness of spreading from cell to cell was evaluated by the ability to form plaques of lysis in the monolayer of bKEC. Bacteria formed visible plaques in the confluent monolayer of cells three days after the infection that suggested their effective spreading from the initially infected cell to neighbors (Figure 5A).

**Figure 6:**
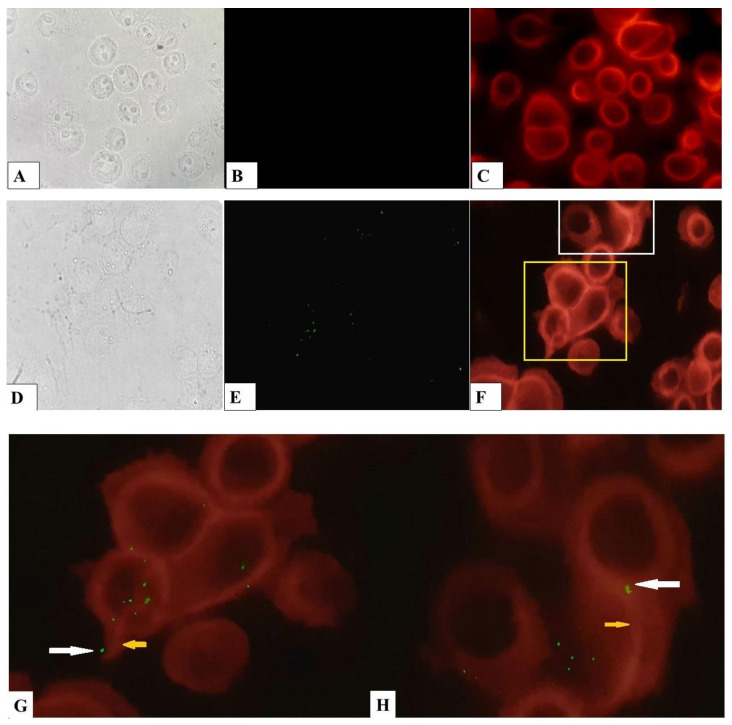
Association with F-actin of *L. monocytogenes* infected bKEC cells. Control (**A–C**) and infected by *L. monocytogenes* (**D–H**); (**A**,**D**) eukaryotic cells in light microscopy (control and infected respectively); (B-control bKEC and E-infected bKEC) cells stained with *L. monocytogenes* antibodies conjugate; (C-control and F-infected bKEC) falloidin labelled F-actin; (**G**,**H**) zoomed areas marked by white- and yellow-framed regions in figure F. Intracellular bacteria associated with falloidin labeled F-actin “comet-tails”, the yellow arrow indicates the area of actin polymerization. The white arrow points to a bacterial cell. The samples were captured with Axio Scope A1 fluorescent microscope at 1000× magnification in three independent replications.

Intracytoplasmic *L. monocytogenes* utilizes host actin filaments to mediate its movement within a cell and from cell to cell [58,59,60]. Actin filaments associated with the bacteria can be visualized by fluorescence microscopy with Alexa Fluor^®^ 555 phalloidin (Thermo Fisher Scientific, Waltham, MA, USA), a reagent that binds to a high-affinity F-actin and not to G-actin. We investigated the ability of *L. monocytogenes* to participate in cytoskeletal rearrangement in bKEC. After 8-h post-infection, we visualized intracellular bacteria and actin microfilaments with fluorescent microscopy. Staining of non-infected cells showed that most of F-actin was concentrated around the nucleus. Cells infected with bacteria also showed a concentric arrangement of F-actin, but it was possible to notice cytoskeleton rearrangements to the “comet-tail” structures associated with bacteria (Figure 6G,H).

### 2.4. Invasion Factors InlA and InlB Are Required for L. monocytogenes Invasion into bKEC

Host cell invasion is mediated by the *L. monocytogenes* surface proteins of the internalin family, InlA and InlB [41,61,62]. The cognitive eukaryotic receptors for InlA and InlB are E-cadherin and c-Met, respectively, which are conserved receptors found on the surface of the epithelial cell [63,64,65,66].

Mechanisms of interactions of InlA and InlB and their target receptors are well-studied. InlA recognizes the human N-terminal immunoglobulin (Ig)-like domain EC1 (amino acid residues 1–95), which is the first of five extracellular Ig-like domains of E-cadherin. InlB interacts with the c-Met PSI- (for plexin-semaphorin-integrin) and Ig1-domains, which are located consecutively in the human c-Met (amino acid residues 519–656) [67,68]. 

To understand the potential of InlA and InlB to interact with bat receptors, we compared internalin-binding domains in human, bat, mouse and guinea pig receptors. The genome of *P. nathusii* is not yet available, so we took sequences of related bat species *Myotis lucifugus* (accession numbers of XP_023611752 and XP_006100243 for E-cadherin and c-Met, respectively) and *Myotis brandtii* (accession numbers of XP_014384634 and XP_005877763 for E-cadherin and c-Met, respectively) belonging to the same family of *Vespertilionidae* available in GenBank. Only E-cadherin EC1-domain and c-Met PSI-Ig1-domains, which are directly involved in interactions with bacterial invasion factors, were compared. 

**Figure 7:**
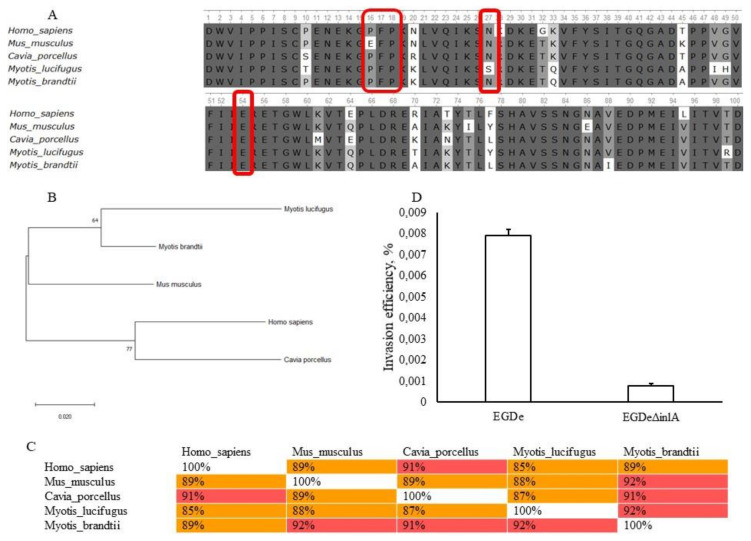
Characteristics of *L. monocytogenes* invasion into bKEC depends on InlA. (**A**) Alignment of protein sequences from human (*H. sapiens*), mouse (*M. musculus*), guinea pig (*C. porcellus*) and bats (*M. lucifugus and M. brandtii*) E-cadherin EC1-domains (performed using the Clustal W program (Conway Institute UCD Dublin by Des Higgins, Dublin, Leinster, Ireland) ). The areas marked in red are amino acids that are necessary for the interaction between E-cadherin and InlA; (**B**) The amino acid sequences were analyzed using the maximum likelihood method in MEGA 7.0 (https://www.megasoftware.net/) using the Jones–Taylor–Tornton (JTT+G) model and with a bootstrapping of 500 replicates. The analysis involved 5 sequences of the EC1-domain. The numbers at nodes represent bootstrap values. Branch lengths are scaled according to the numbers of amino acid substitutions per site; (**C**) To assess the matching of the distance between multiple sequence alignments we have utilized Unipro UGENE software (http://ugene.net/); (**D**) Invasion efficiency test in bat kidney cells by the wild type strain EGDe and its derivative EGDeΔ*inlA* lacking the *inlA* gene. The mean ± SD from three independent experiments are shown.

The maximum likelihood dendrogram and the multiple alignment sequence scores revealed that the bat E-cadherin EC1-domain is less similar to human E-cadherin than mouse and guinea pig proteins (Figure 7A,B). EC1-domain interacts with InlA with the highest value being proline at position 16 of EC1-domain [67,69]. The alignment analysis showed that in bats, as well as in humans and guinea pigs, proline is located in the 16 position. This is in contrast to mouse E-cadherin, for which proline 16 is replaced by glutamine that prevents interactions of InlA with mouse E-cadherin (Figure 7A) [69]. Other residues important for E-cadherin and InlA interactions, including Phe17, Pro18, Asn27, and Gly54 [67], were conserved among sequences of *Homo sapiens, Cavia porcellus, Mus musculus* and *M. brandtii*. For *M. lucifugus*, the substitution Asn27Ser was revealed. However, this substitution can be considered as synonymous as both asparagine and serine are hydrophilic and have a similar charge. Therefore, despite the relatively low similarity of the bat and human EC1-domains (Figure 7C), the bat E-cadherins carries key amino acid residues required for interactions with InlA (Figure 7A).

To test this assumption, we compared invasion efficiency of the wild type strain EGDe and its isogenic deviation, EGDeΔ*inlA*, with the deletion of the *inlA* gene. Invasion efficiency of EGDeΔ*inlA* was 0.00077 ± 0.00002%, i.e., 10-fold less than the invasion efficiency of EGDe (*p* < 0.000001, Figure 7D). These data support our suggestions that *L. monocytogenes* invasion in bKEC depends on InlA.

**Figure 8:**
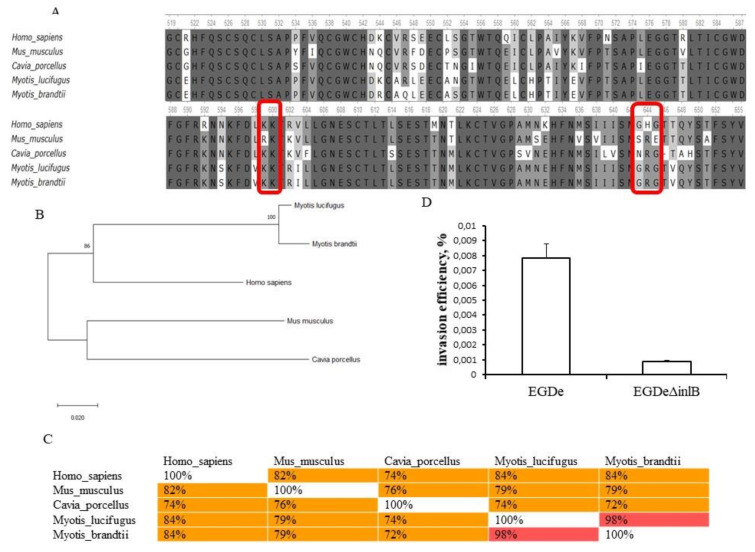
Characteristics of *L. monocytogenes* invasion into bKEC depends on InlB. (**A**) Alignment of protein sequences from human (*H. sapiens*), mouse (*M. musculus*), guinea pig (*C. porcellus*) and bats (*M. lucifugus and M. brandtii*) c-Met PSI-Ig1-domains (amino acids 519–659 of human Met 1–95) (performed using the Clustal W program (Conway Institute UCD Dublin by Des Higgins, Dublin, Leinster, Ireland). The areas marked in red are amino acids that are necessary for the interaction between c-Met and InlB; (**B**) The amino acid sequences were analyzed using the maximum likelihood method in MEGA 7.0 (https://www.megasoftware.net/) using the Jones–Taylor–Tornton (JTT+G) model and with a bootstrapping of 500 replicates. The analysis involved 5 sequences of the c-Met PSI-Ig1-domains. The numbers at nodes represent bootstrap values. Branch lengths are scaled according to the numbers of amino acid substitutions per site; (**C**) To assess the matching of the distance between multiple sequence alignments we have utilized Unipro UGENE software (http://ugene.net/); (**D**) Invasion efficiency of the wild type strain EGDe and its derivative EGDeΔ*inlB* lacking the *inlB* gene. The mean ± SD from three experiments are shown.

Next, we analyzed the importance of another invasion factor, InlB, for *L. monocytogenes* invasion into bKEC cells. The concave face of the InlB, leucine-rich repeat region, interacts tightly with the PSI-Ig1-domains of the c-Met stalk [68]. The partial sequence of bat c-Met was aligned with the human, guinea pig, and mouse c-Met proteins (Figure 8A). The amino acids in the Ig1-domain involved in interactions of human c-Met with InlB, including lysines Lys599 and Lys600, and glycines Gly643 and Gly645 (Figure 8A) [68], were conserved in bats. In the guinea pig, Gly643 was missing that might explain the inability of InlB to interact with guinea pig c-Met. Taken together, these results showed that bat c-Met carries all key amino acid residues and seemed to be able to interact with InlB.

The maximum likelihood dendrogram showed that the PSI-Ig1-domains of the bat species *M. lucifugus and M. brandtii* formed the cluster together with human c-Met (Figure 8B). The similarity of bat and human proteins was 84 % that was higher than for human and mouse, and for human and guinea pig sequences (82 % and 74 %, respectively) (Figure 8C). The similarity between bat proteins was 98% that was higher than its similarity to c-Met proteins of other species (Figure 8C). 

To determine the role of InlB in interactions of *L. monocytogenes* with bat cells, bKEC was infected with the isogenic strains EGDe and EGDeΔ*inlB* (Figure 8D). Invasion efficiency of EGDeΔ*inlB* was 0.00031 ± 0.000011%, i.e., 25-fold less than the invasion efficiency of EGDe (*p* < 0.000001). The lack of InlB resulted in a very low invasion by single bacterial cells. Therefore, both InlA and InlB are involved in *L. monocytogenes* invasion into bat cells.

### 2.5. Natural InlB Variants Differently Restored Invasion of the Strain EGDeΔinlB.

**Figure 9:**
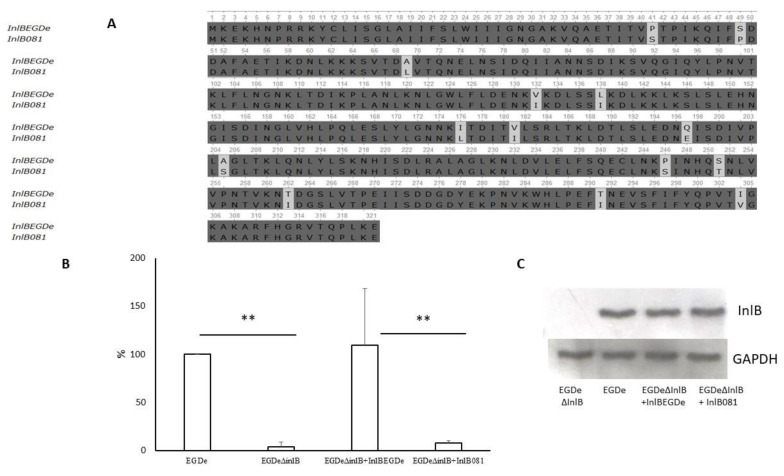
Natural InlB variants restored invasion of the strain EGDeΔ*inlB*. (**A**) Alignment of InlB protein sequences from *L. monocytogenes* (performed using the Clustal W program by UGENE (http://ugene.net/); (**B**) Impact of InlB on invasion efficiency in bat kidney epithelial cells. Cells were infected with an MOI of 100, and the invasion efficiency is shown as the ratio of the number of intracellular bacteria of a mutant strain to that of strain EGDe. Data shown are expressed as mean ± SD from three experiments performed in triplicate. Statistical significance of competition experiments relative to a corresponding positive control is shown (** *p* < 0.01); (**C**) Immunoblotting analysis of membrane-bound proteins of the parental strain EGDeΔ*inlB*, EGDe and recombinant strains encoding InlB variants. GAPDH of *L. monocytogenes* was used as a control.

The deletion of the *inlB* gene almost completely disrupted *L. monocytogenes* invasion into bKEC cells, providing a higher effect than the deletion of the *inlA* gene (25-fold and 10-fold respectively). To get a better understanding of the role of InlB in bat cell invasion, we supplemented the *inlB* deletion with two distinct *inlB* alleles and compared the invasion of the recombinant strains into bKEC cells. One *inlB* allele designated as *inlB*_EGDe_ was cloned from the strain EGDe and was expected to restore invasion. Another allele designated as *inlB*_081_ was from the *L. monocytogenes* strain VIMVR081, isolated from the liver of the grey red-backed vole (*Myodes rufocanus*) captured in the pristine environment in the far eastern part of Eurasia [26]. The strain VIMVR081 (accession number NZ_CP018148.1) belongs to the clonal complex CC2 [30]. Results of MLST typing are available from the *L. monocytogenes* Institut Pasteur MLST database at http://www.pasteur.fr/mlst and GeneBank database at https://www.ncbi.nlm.nih.gov/assembly/GCF_001889585.1. The same *inlB* allele was found in wild rodent isolates belonging to the clonal complexes CC2 and CC315 [26,30]. The *inlB* alleles were cloned into the pTRKH2 vector as described in Materials and Methods. The c-Met binding domains of the recombinant proteins differed by 14 amino acid substitutions (Figure 9A). The obtained strains were used to infect bKEC cells as described above. The allele *inlB*_EGDe_ as it was supposed, restored invasion (Figure 9B). The bacterial strain carrying the *inlB_081_* allele had low efficiency in invasion. The invasion efficiency of the strain carrying *inlB*_081_ was only two-fold higher than that of the strain EGDeΔ*inlB* (*p* < 0.05). The level of InlB expression in the EGDe strain and recombinant strains was at the same level (Figure 9C). Obtained data supported the view that InlB is important for *L. monocytogenes* invasion into bat cells and suggested the importance of the InlB isoform for bat cell invasion.

## 3. Discussion

*Chiroptera* is the most numerous (majority) group of mammals that includes up to a quarter of all known mammalian species representing one of the most abundant animal species on the planet [18]. Bats can actively occupy anthropogenic environments, increasing risks of infection transmission to human populations. The Covid19 epidemic is an example of the potential role of bats as a source of dangerous infections for humans. [3,70]. 

In this study, we performed in vitro testing of bat susceptibility to the pathogenic bacterium *L. monocytogenes* belonging to the group of infectious agents with a sapronotic origin, also known as soilborne pathogens [71,72]. This large group of microorganisms includes human pathogens such as *L. monocytogenes, Y. pseudotuberculosis*, *Legionella pneumophila*, *Francisella tularensis*, and *Pseudomonas aeruginosa* [8]. These pathogenic bacteria circulate in natural environments and can be transmitted to anthropogenic environments, usually by wild carriers. The typical feature of such pathogens is polyhostality, i.e., the ability to cause disease in a wide range of susceptible hosts. Polyhostality can be well illustrated by *L. monocytogenes*, which is a proven pathogen of humans and domestic animals [24]. 

To prevent the transition of new pathogens from natural hosts to humans, understanding the mechanisms that underlie pathogen polyhostality is required, but this is impossible without experimental studies on interactions of the pathogen with different groups of hosts. However, the use of laboratory animal models can be difficult, both from the ethical point of view and because of the specific physiology of the object under study: most bats live in colonies including enormous numbers of individuals, and their body temperatures may physiologically vary between less than 20 °C during hibernation and more than 40 °C during flight [73]. To solve the ethical issues and accumulate primary data on host–pathogen interactions on a molecular basis, it is beneficial to use cellular models. To be useful, a cellular model should demonstrate the reliable stability of basic cellular characteristics.

In our study, a finite culture of kidney cells (bKEC) was obtained for the first time from the widespread small bat Nathusius’ pipistrelle. Cytogenetic analysis of bKEC cell culture revealed a diploid modal number of 2n = 44 and a total number of chromosomal arms of FN = 50; these findings are consistent with the data obtained for some other representatives of the genus *Pipistrellus*, such as *P. savii koreensis* and *P. kuhli* [74,75,76]. The bKEC culture demonstrated high stability, and its morphological and karyotypic characteristics remained stable from the 3rd up to the 36th consecutive re-plating steps. This stability provided reliable results on interactions between bKEC cells and the facultative intracellular pathogen *L. monocytogenes*.

Our data demonstrate the ability of wild-type *L. monocytogenes* once inside bat cells. The observed level of invasion was 0.0078 ± 0.0009% or about 301 ± 11 bacterial cells per well. This relatively low invasion efficiency is consistent with the results of other authors that used primary or finite cell lines of human, mouse and pig origin [40,41]. Immortalization of finite cell lines results in a noticeable increase in the *L. monocytogenes* invasion [77]. Finite pig and mouse cells showed a level of invasion by *L. monocytogenes* that was approximately two orders magnitude less than that of continuous cell lines obtained from the finite cells by transformation with the SV40 virus [78]. 

Once entered bat cells, *L. monocytogenes* doubled its number within 70 min. The doubling time of 70 min is consistent with the results for the human intestinal epithelial cell line Henle 407, in which *L. monocytogenes* had a doubling time of approximately 60 min [79]. The generation time in human enterocyte-like CaCo-2 cells is about 90 min, while in Madin–Darby canine kidney epithelial cells, it reaches 180 min [80,81]. The obtained results suggest that bKEC cells effectively support *L. monocytogenes* multiplication.

Entrance to and multiplication within epithelial cells are critical steps of *L. monocytogenes* infection in mammals, particularly required for crossing the intestinal barrier, followed by entering the lamina propria and intestinal lymph nodes [53]. The obtained data on the successful interactions of *L. monocytogenes* with bat epithelial cells are in line with the previously reported *L. monocytogenes* isolation from bat intestinal lymph nodes [14]. 

The data obtained in this study suggest that the initial stages of listeriosis in humans and bats are similar, at least when it comes to the need for InlA and InlB [41,82]. For comparison, in mice and guinea pigs, the invasion of epithelial cells depends on only one of the two invasion factors, either InlB or InlA, respectively [54,69]. Our previous findings have demonstrated that naturally occurring variants of InlB domain differ from each other on their ability to support intragastric infection in mice [50]. InlB variant of CC2 was found in 13 phylogenetically distant *L. monocytogenes* isolated from wild small rodents in remote parts of Russia in different years [30]. However, the distribution of this variant was not restricted to rodent isolates [30]. We used CC2 variant InlB to compare invasion of *L. monocytogenes* in mouse cells and bat cells. Interestingly, the InlB variant found in *L. monocytogenes* CC2 strains isolated from wild small rodents was less effective in bat cells than the InlB variant from the strain EGDe (Figure 9B). To explain this effect, further investigations are needed. 

Taken together, the obtained data suggest that bats represent another phylum of widespread small mammals that support *L. monocytogenes* cell invasion via mechanisms similar to those active in humans. We believe that the cell model developed in this work will be useful for in vitro studies of intracellular bacteria and will help to provide insight into the mechanisms of the polyhostality of *L. monocytogenes* and other sapronotic pathogens.

## 4. Materials and methods

### 4.1. Animals

All the experiments on animals were performed in accordance with Russian legislation on animal welfare, which is in line with the Directive of the European Parliament 2010/63/EU, Code of ethics for the conduct of biomedical research with the use of animals, documents of the International UNESCO bioethics Committee, intergovernmental Committee UNESCO bioethics, and Steering Committee on bioethics of the Council of Europe, with the approval of FRCVM Bioethics commission control (Protocol #2020-01/1).

Nathusius’s pipistrelle bats (*P. nathusii)* were caught using a fog net in the Volgograd region, Russia. Two clinically healthy male individuals weighing 15 and 12 grams were selected as kidney donors. Bats were euthanized using an intraperitoneal injection of 500 mL of 70% ethanol and dislocation of the cervical vertebrae after two minutes of post-cardiac arrest.

### 4.2. Tissue Trypsinization and Cell Isolation 

In the first step, we removed the renal pelvis and the medulla from the kidneys. Then, the cortical layer of the kidney was mechanically ground to fragments with sizes of about 2–3 mm^3^ in 50 mL sterile tubes (Eppendorf, Hamburg, Germany). Pieces of the tissue were washed five times with MEM (HyClone, Marlborough, MA, USA) to remove blood. Then, fragments of bat kidneys were transferred to a flat-bottomed flask with a volume of 1.0 dm^3^ filled with a dispersing solution of 0.25% trypsin (PANEKO, Moscow, Russia) and 0.02% versene (PANEKO, Moscow, Russia) in the proportion of 2:1 and pre-warmed to 37 °C. The tissues were mixed on a magnetic stirrer for 15 min. Then, the flask was removed from the magnetic stirrer, non-disrupted tissue pieces were allowed to settle for 2 min, and the cell suspension was transferred into a 0.5 L vial. Then, FBS (HyClone, Marlborough, MA, USA) was added to the cells in a dispersing solution to neutralize trypsin up to 10%. The non-disrupted tissue pieces were reprocessed in a new cycle of enzymatic degradation with stirring. The procedure was repeated three times until complete tissue depletion. Bat cells were harvested by centrifugation at 1000 rpm for 10 min. The sediment of cells was resuspended in 25 mL of the MEM medium with the addition of 10% of FBS. The derived cell suspension was filtered through a gauze filter designed for the mechanical elimination of non-trypsinized fragments of renal tissue. Estimation of the viability of cells performed in a trypan blue (Thermofisher Scientific, Waltham, MA, USA) vital staining test revealed that it was 78%.

In the second step, the obtained cell suspension was diluted with DMEM (HyClone, Marlborough, MA, USA) supplemented by 10% FBS, 10 µg/mL ciprofloxacin (Sintez, Kurgan, Russia), and 5 µg/mL amphotericin B (Sintez, Kurgan, Russia) to achieve a seeding density of 4 × 10^5^ cells/mL. This cell suspension was transferred into plastic cell culture flasks (Corning, USA) and incubated for 3 h to allow cells to attach. The cells were incubated at 37 °C with 5% CO_2_. Finally, the flasks were carefully washed with the DMEM medium to remove non-attached cells and further cultivated in the same medium.

The growth medium was changed after the first day of cultivation and then every 2–3 days. The growth intensity and cytomorphological characteristics of cells were evaluated using an inverted microscope (Olympus CK2, Tokyo, Japan). The homogenous culture of epithelial cells obtained at the second passage was named the bKEC culture.

### 4.3. Cell Culture and Subculture

bKEC cells were inoculated into a 24-well plate (Corning, Corning, NY, USA) and incubated at 37 °C in a 5% CO_2_ atmosphere. After adhesion, the DMEM medium was changed, and the plates were examined daily under an inverted microscope (Olympus CK2, Tokyo, Japan) to estimate the cell growth. Subculturing was performed by successive passages in DMEM medium.

### 4.4. Cryopreservation and Thawing of Cells

A cryobank of cell subcultures was created. Subcultures obtained at the 4th, 10th, 13th, 18th, 20th, 22nd, 28th and 33rd passages were cryopreserved in liquid nitrogen. Cryopreservation of cells was performed in sterile DMEM medium with the addition of 10% DMSO (Sigma-Aldrich, St. Louis, MO, USA) as a cryoprotector after equilibrating at 4 °C for 60 min, followed by rapid freezing at −70 °C [83]. After 14 days of storage, frozen cells were deep-frozen in liquid nitrogen (−196 °C). Immediately before the experiment, cryovials with cells were thawed at 37 °C in a water bath. Then, the contents of the cryovials were added to a sterile Falcon tube containing DMEM medium, warmed up to 37 °C, and centrifuged at 1000 rpm for 10 min. The cell pellet was resuspended in DMEM medium, transferred into new vials, and incubated at 37 °C in a 5% CO_2_ atmosphere [84]. 

### 4.5. Chromosome Preparations and Karyological Analysis

The daily culture of bKEC cells was used for chromosome preparations. Karyological analysis was carried out according to the standard technique [85,86].

### 4.6. Bacterial Strains and Growth Conditions

The wild-type *L. monocytogenes* strain EGDe and its derivatives EGDeΔ*inlA* and EGDeΔ*inlB*, lacking the *inlA* and *inlB* genes, respectively, were used [87]. The strains with *inlA* and *inlB* deletions were generously provided by Prof. J. Vazquez-Boland, Univ. Edinburgh, UK, and have been used in previous studies [50,88]. Complementation of the *inlB* deletion with the InlB-expressing plasmid was described earlier [50]. Briefly, the *inlB* gene was expressed from the promoter of the *inlAB* operon cloned into the shuttle vector pTRKH2 [50,89]. To maintain the plasmid, erythromycin (Sigma-Aldrich, St. Louis, MO, USA) was added to the medium to a concentration of 10 µg mL^−1^.

*L. monocytogenes* was cultivated in the BHI medium (Becton, Dickinson and Company, East Rutherford, NJ, USA) and grown at 37 °C with agitation at 180 rpm. Plasmid-bearing strains were grown in the presence of 10 µg/mL erythromycin. To prepare a culture for infection, bacteria were grown to the mid-exponential phase, washed with PBS (Amresco, Solon, OH, USA) three times, aliquoted, and frozen in the presence of 10% glycerol (Sigma-Aldrich, St. Louis, MO, USA). The use of pre-frozen cultures allows for more accurate standardization of infection doses between the compared strains. The concentration of bacterial cells was determined by plating serial dilutions from the frozen culture the day before the experiment.

### 4.7. In Vitro Invasion and Proliferation Assay

bKEC cells from the fourth passage of subcultures were used in the experiment. Before the analysis of invasion and proliferation, bKEC cells were cultured without antibiotics for 5 days. An invasion assay was performed according to Suarez et al. [90]. Bacteria frozen in 10% glycerol were thawed immediately before the experiment and resuspended in PBS to a volume of 1 mL. The bacterial suspension was diluted in DMEM with 10% FBS to achieve final concentrations with a multiplicity of infection (MOI) of 100 CFU per cell and added to the cells reached 70–80% confluence in a 24-well. After 1 h incubation at 37 °C in a 5% CO_2_ atmosphere, cells were washed with PBS three times, and fresh DMEM supplemented with 100 µg mL^−1^ gentamicin (Sigma-Aldrich, St. Louis, MO, USA) was added to kill extracellular bacteria. After 1 h of contact with gentamicin, cells were washed with PBS and incubated with 200 µL of the mixture of 0.25% trypsin and 0.02% EDTA (Sigma-Aldrich, St. Louis, MO, USA) in the proportion of 2:1 for 10 min. Then, 800 µL of PBS was added to each well, and cells were collected in centrifuge tubes (SSIbio, Lodi, CA, USA) and treated with ultrasound at 18 kHz for 10 s in an ice bath. The number of bacteria that successfully entered cells was assessed by plating the serial dilutions of cell lysates on a solid BHI medium. Plates were incubated at 37 °C for 24 h, and then colonies were quantified. The efficiency of invasion was evaluated by the ratio of the number of entered bacteria to that of bacteria used for cell infection.

To assess intracellular proliferation, infected cells were incubated in the gentamicin-containing medium at 37 °C. Cells were lysed at 2, 4, 6 and 8 hpi (hours post-infection), and bacteria were plated from lysates as described above. The effectiveness of intracellular proliferation was evaluated by determining the number of CFU at this time to the number of introduced bacteria. All the experiments were performed in triplicate and repeated at least three times.

### 4.8. Sequence Analysis 

The sequences of proteins were compared with those available in GenBank using Basic Local Alignments Tool (BLAST) analysis. For c-Met sequence were used accession numbers XP_006100243, XP_005877763, NM_000245.4, NP_032617.2 and XP_003475185.1 and for EC1-domain of E-cadherin we used accession numbers XP_023611752, XP_014384634, NP_004351.1, NP_033994.1 and XP_005004692.1. Sequences were proofread and assembled in Unipro UGENE version 35 (http://ugene.net/). Protein alignment was performed using Clustal W. To assess the matching of the distance between multiple sequence alignments we have utilized Unipro UGENE software (http://ugene.net/). The evolutionary history was inferred by Maximum Likelihood method based on the Jones–Taylor–Thornton (JTT+G) substitution model (500 bootstrap cycles) [91]. Dendrograms were constructed with Mega 7.0 (https://www.megasoftware.net/) by the method proposed by Kumar et al. [92]. Allelic numbers of InlB were determined using the *L. monocytogenes* MLST database (https://bigsdb.pasteur.fr/listeria/listeria.html).

### 4.9. Immunoblotting 

The restoration of InlB expression was checked by sodium dodecyl sulfate-polyacrylamide gel electrophoresis (SDS-PAGE). Membrane-bound protein samples were prepared from overnight *L. monocytogenes* cultures grown in BHI broth supplemented with 0.2% charcoal to activate the PrfA regulon as described previously [93]. SDS-PAGE was performed in accordance with the generally accepted techniques. Cell lysates of *L. monocytogenes* were boiled for 10 min, separated on 10% SDS-PAGE and transferred onto PVDF membrane. InlB was visualized with polyclonal primary anti-InlB antibodies (were obtained as described in the next section) and donkey anti-rabbit IgG HRP-labeled antibodies (Abcam, London, UK).

### 4.10. Primary Antibody Development and Purification

Two male rabbits (weighing 2.5 kg) were immunized with the recombinant protein InlB321/15 according to the scheme (Table 1). InlB321/15 was purified from the recombinant *E. coli* BL21::pET28b::inlBallele9 strain as described previously [94]. Briefly, after the first immunization with the protein applied intravenously, subcutaneously and intramuscularly injections were performed on the 4th, 8th and 10th weeks. Blood samples were taken after two weeks post the last immunization and onwards every two weeks for 3 months long. Blood samples were collected in vacuum tubes Improvacuter^®^ with coagulation activator (SiO_2_) and gel (Guangzhou Improve Medical Instruments CO., LTD., Guangzhou, China). After coagulation, the blood was centrifuged at 3000 rpm for 15 min. Sera were stored at −20 °C. Anti-InlB_321_ antibodies were purified from the antiserum using an immunosorbent. The immunosorbent developed on the basis of BrCN-activated sepharose 4B (Pharmacia, Srockholm, Sweden) conjugated with purified InlB_321_ according to the manufacturer’s instructions. Immunoadsorption was carried out in phosphate buffer (pH 7.0) with 0.15 M NaCl. After applying the antiserum, the column was washed with a phosphate buffer (pH 7.0) with 0.3 M NaCl. Antibody elution was performed with 4.5 M MgCl_2_ followed by a dialysis step. Antibodies were stored in 50% glycerol at −80 °C. Anti-GAPDH-antibodies were obtained analogically.

### 4.11. Plaque Forming Assay

Bacteria for the assay were prepared and used for the infection of the confluent monolayer of bKEC cells cultured in six well plates. The assay was performed in general as described in [95]. After 1 h incubation, the cells were washed three times with PBS, and non-penetrated bacteria were killed by 1 h incubation with gentamicin (100 μg mL^−^^1^). The infected monolayers were then overlayed with 10 mL of the soft agar, prepared by mixing equal volumes of 2 × DMEM supplemented with 20 μg mL^−1^ gentamicin and 2% agar. Cells were further incubated under 5% CO_2_ at 37 °C for up to three days following infection, and plaques were visualized by staining monolayers with 1 mL Neutral Red Solution (Sigma Aldrich, St. Louis, MO, USA) diluted 1:10 in PBS for 3 h. A culture of non-infected cells was used as a control.

### 4.12. Determination of Cytoskeleton Rearrangements and Bacterial Cells Visualization in bKEC

The bKEC cells were streaked on cover glasses at a concentration of 10,000 cells/cover glass and incubated in the DMEM medium with 10% FBS for 18 h. Next, we did the infection as described in the “Materials and Methods” section. The negative control was the non-infected cells. After 8-h incubation, the cells were washed two times in PBS and fixed with 3.7% formalin for 10 min. The cells were then permeabilized with 0.1% Triton X-100 (Panreac, Barcelona, Catalonia, Spain) for 10 min and stained with *L. monocytogenes* antibody-fluorescein isothiocyanate conjugate (FITC) (LifeSpan BioSciences, Seattle, WA, USA) Inc. for 1 h. Then cells washed three times with PBS and stained with Phalloidin Alexa Fluor 555 (Thermo FisherScientific, Waltham, MA, USA) for 20 min as described by the producer. All samples were assessed and captured thrice with Axio Scope A1 fluorescence microscope at 1000× magnification.

### 4.13. Statistics 

Student’s unpaired *t*-test, included in the Excel software package (Microsoft, Redmond, WA, USA), was used, and *p*-values of less than 0.05 were considered statistically significant.

## Figures and Tables

**Table 1:** Immunization scheme.

Immunization	Week	Dose (mg)	Adjuvant	Mode of Administration
1st	0	0.5	-	Iv
		0.375	CFA	Sc
		0.375	CFA	Im
2nd	4	0.25	-	Iv
		0.75	IFA	Sc
3rd	8	0.25	-	Iv
		0.75	IFA	Sc
4th	10	0.25	-	Iv
		0.75	IFA	Sc
	12	Sampling		

Note: Iv, intravenous; Im, intramuscular, in a leg; CFA, complete Freunds adjuvant; Sc, subcutaneous, in a back; IFA, incomplete Freunds adjuvant.
